# Natural killer cells induce HIV-1 latency reversal after treatment with pan-caspase inhibitors

**DOI:** 10.3389/fimmu.2022.1067767

**Published:** 2022-12-06

**Authors:** Joana Furtado Milão, Luca Love, George Gourgi, Lukas Derhaschnig, J. Peter Svensson, Anders Sönnerborg, Robert van Domselaar

**Affiliations:** ^1^ Division of Infectious Diseases, ANA Futura Laboratory, Department of Medicine Huddinge, Karolinska Institutet, Stockholm, Sweden; ^2^ Department of Biosciences and Nutrition, Karolinska Institutet, Stockholm, Sweden; ^3^ Division of Clinical Microbiology, ANA Futura Laboratory, Department of Laboratory Medicine, Karolinska Institutet, Stockholm, Sweden

**Keywords:** caspases, HIV, latency, NK cells, reactivation, shock-and-kill

## Abstract

The establishment of a latency reservoir is the major obstacle for a cure of HIV-1. The shock-and-kill strategy aims to reactivate HIV-1 replication in HIV -1 latently infected cells, exposing the HIV-1-infected cells to cytotoxic lymphocytes. However, none of the latency reversal agents (LRAs) tested so far have shown the desired effect in people living with HIV-1. We observed that NK cells stimulated with a pan-caspase inhibitor induced latency reversal in co-cultures with HIV-1 latently infected cells. Synergy in HIV-1 reactivation was observed with LRAs prostratin and JQ1. The supernatants of the pan-caspase inhibitor-treated NK cells activated the HIV-1 LTR promoter, indicating that a secreted factor by NK cells was responsible for the HIV-1 reactivation. Assessing changes in the secreted cytokine profile of pan-caspase inhibitor-treated NK cells revealed increased levels of the HIV-1 suppressor chemokines MIP1α (CCL3), MIP1β (CCL4) and RANTES (CCL5). However, these cytokines individually or together did not induce LTR promoter activation, suggesting that CCL3-5 were not responsible for the observed HIV-1 reactivation. The cytokine profile did indicate that pan-caspase inhibitors induce NK cell activation. Altogether, our approach might be–in combination with other shock-and-kill strategies or LRAs–a strategy for reducing viral latency reservoirs and a step forward towards eradication of functionally active HIV-1 in infected individuals.

## Introduction

HIV-1 infection remains a major health challenge worldwide and no cure is available despite efficient antiretroviral therapy (ART). The innate and adaptive immune systems together with ART control the infection but fail to eliminate the virus from the host. The viral latent reservoir consists of CD4^+^ T lymphocytes and macrophages carrying an integrated replication-competent copy of the HIV-1 genome that is silenced but can become reactivated leading to reappearance of viremia (1). Integrated provirus can be silenced through epigenetic mechanisms, for example through HP1γ or the human silencing hub complex (2, 3). Although the return of CD4^+^ T lymphocytes to their resting stage induces latency, HIV-1 silencing can also happen stochastically (4, 5). Reversing HIV-1 latency reactivates proviral transcription mediated by activating transcription factors and epigenetic modulation. The U3 region of the HIV-1 long terminal repeat (LTR) promoter contains binding sites for several transcription factors: NF-kB, NFAT, Sp1, LEF, Ets-1, and USF1. Thus, biological or chemical agents that lead to the activation of these transcription factors could result in HIV-1 reactivation within latently infected cells.

The shock-and-kill strategy aims to reactivate viral transcription of the integrated HIV-1 genome in latently infected cells (6, 7). The reactivated cells will become exposed to immune cells and susceptible to cell death induced by cytotoxic lymphocytes and/or cytopathic effects derived from viral replication. This selective cell death should reduce the latent viral reservoir over time and eventually result in the eradication of HIV-1. However, many details of the latent HIV reservoir remain unknown, including what cell types make up the latent reservoir, the size of the reservoir, and its anatomical locations (8). To date, no biomarker has been identified that discriminate HIV-1 latently infected cells from healthy uninfected cells. Therefore, the currently developed latency reversal agents (LRAs), such as protein kinase C agonists, pattern recognition receptor agonists or immune checkpoint inhibitors, have to act systemically in order to reach the viral reservoirs but without causing severe side-effects. Although LRAs show promise in *in vitro* and animal models, a significant reduction in the latency reservoir within people living with HIV-1 has not yet been achieved (9–12). Adaptations or combinations of the shock-and-kill strategy might be required to achieve substantial reductions of the viral latency reservoirs within people living with HIV-1.

The shock-and-kill strategy also relies on cell-mediated cytotoxicity by cytotoxic lymphocytes for the “kill” ability. Cytotoxic lymphocytes, *e.g.*, natural killer (NK) cells and CD8^+^ T lymphocytes, are key components of both the innate and adaptive immune responses to control virus infections. These cells can eliminate virus-infected cells through the induction of target cell death *via* multiple mechanisms (13–15). In a previous study, we showed that NK cells target the HIV-1 Gag protein through the release of protease granzyme M (GrM) (16). Because GrM is known to inhibit replication of other viruses (17–19), we were initially interested in whether NK cells could also inhibit HIV-1 replication in a GrM-dependent manner. Unexpectedly, however, when assessing this in a co-culture assay, we observed that NK cells can obtain the “shock” ability towards HIV-1 latently infected cells when the NK cells were treated with pan-caspase inhibitors. We further assessed how NK cells could mediate this HIV-1 latency reversal, including analysing changes in cytokine secretion after treatment with pan-caspase inhibitors in different latency models. Our *in vitro* data suggests that NK cells might be used in an indirect shock-and-kill strategy instead of directly targeting the latently infected cells.

## Materials and methods

### Cell culture

Cells were cultured in 5% CO_2_ at 37°C. TZM-bl cells were maintained in Dulbecco’s modified Eagle medium (DMEM, Gibco/ThermoFisher Scientific, Waltham, MA, USA) supplemented with 10% fetal bovine serum (FBS, Sigma/Merck, Darmstadt, Germany), 2 mM L-glutamine (Sigma), 0.1 mM MEM Non-Essential Amino Acids (Gibco), and 20 units/mL penicillin combined with 20 μg/mL streptomycin (Sigma). KHYG-1 cells (#ACC 725, DSMZ, Braunschweig, Germany) were maintained in Roswell Park Memorial Institute 1640 (RPMI, Sigma) medium supplemented with 10% FBS, 25 mM HEPES, 20 units/mL penicillin and 20 μg/mL streptomycin, and 100 units/mL of recombinant human interleukin-2 (IL-2, PeproTech, Cranbury, NJ, USA). J-Lat 10.6 cells were maintained in RPMI medium supplemented with 10% FBS, 25 mM HEPES, 20 units/mL penicillin and 20 μg/mL streptomycin. The J-Lat 1C10 cell line construction is described in (20). J-Lat 1C10 cells were cultured in cytokine-free media (RPMI 1640 medium, Hyclone/Cytiva, Marlborough, MA, USA), 10% FBS (Life Technologies), 1% Glutamax (Life Technologies), and 1% Penicillin-streptomycin (Life Technologies).

### Reagents

Pan-caspase inhibitor Z-VAD-FMK and individual caspase inhibitors (Caspase-Family Inhibitor Set IV) were purchased from Enzo Life Sciences (Farmingdale, NY, USA) and GrM inhibitor Ac-KVPL-CMK from Peptanova (Sandhausen, Germany). Pan-caspase inhibitors Q-VD-Oph and emricasan were purchased from Selleckchem (Houston, TX, USA). CellTrace™ Violet proliferation kit, LIVE/DEAD Fixable Violet Dead Cell Stain Kit and LIVE/DEAD Fixable Near IR Dead Cell Stain Kit were obtained from ThermoFisher Scientific. Flow cytometry antibodies CD3-PE (clone OKT3), CD16-PerCP-Cy5.5 (clone 3G8), and CD56-PE-Cy7 (clone HCD56) were obtained from Biolegend (San Diego, CA, USA). CCR5-BV421 (clone 2D7) and its isotype control (mouse C57BL/6 IgG2a, κ, BV421) were obtained from BD Biosciences (Franklin Lakes, NJ, USA).

Recombinant human IL-21, recombinant human IL-10, recombinant human MIP-1α (CCL3), recombinant human MIP-1β (CCL4), recombinant human RANTES (CCL5) were purchased from PeproTech. Prostratin, phorbol 12-myristate 13-acetate (PMA), and ionomycin were purchased from Sigma. JQ1 was purchased from Cayman Chemical (Ann Arbor, MI, USA) and romidepsin from Celgene (Brystol Meyers Squibb, Summit, NJ, USA).

### Stimulation with (pan-)caspase inhibitors

Seeded KHYG-1 cells, enriched primary NK cells, or J-Lat cells were incubated at 37°C for the times indicated with 50 µM of the pan-caspase inhibitors (10 mM stock solutions in DMSO), 10 µM of individual caspase inhibitors (stock solution is 2 mM in DMSO) from the Caspase-Family Inhibitor Set IV that also contains the pan-caspase inhibitor control Z-VAD-FMK and cysteine cathepsin inhibitor Z-FA-FMK (negative control for FMK inhibitors), or only DMSO (0.5%) as a control. For collection of supernatants, cells were collected, centrifuged at 1500 rpm for 5 min and supernatant was collected. Fractionating of supernatants was performed using Amicon Ultracel Centrifugal Filter columns (3 kDa MWCO, 2 mL sample volume, Millipore/Sigma) according to manufacturer’s protocol with centrifugation at 4000 rpm for 60 min. For the unconcentrated >3 kDa fraction, 10% FBS was added, whereas the concentrated <3 kDa fraction was resuspended in serum-free medium to its original volume.

### (Co-)culture assays

For the co-culture assay, J-Lat 10.6 cells were labelled with 2 µM CellTrace Violet dye on day one. On day two, 10x10^6^ J-Lat were collected and half was stimulated with 6 µM of prostratin for 4 h at 37°C, while the other half was left unstimulated as a control. Cells were washed and resuspended to a concentration of 0.5x10^6^ J-Lat cells/mL and incubated with z-VAD-FMK for 1 h at 37°C. After this incubation period, a cell suspension of 0.5x10^6^ KHYG-1 cells or only medium were added to the J-Lat cells in an effector:target ratio of 1:1. Z-VAD-FMK or DMSO were added to adjust the concentration and cells were incubated overnight in the same conditions as KHYG-1 are maintained, including 100 U/mL of rhIL-2.

For the incubation of J-Lat cells with supernatants of KHYG-1 cells, a cell suspension of 1x10^6^ J-Lat cells was incubated with 6 µM of prostratin for 4 h at 37°C or left unstimulated as a control. J-Lat cells were then washed twice with PBS and resuspended to a concentration of 0.5x10^6^ J-Lat cells/mL using supernatant from KHYG-1 cells or only complete RPMI with DMSO (0.5%) or Z-VAD-FMK (50 µM) and incubated overnight at 37°C.

For the synergy experiments, 0.5x10^6^ J-Lat cells were resuspended in 0.5 mL complete RPMI or supernatants from treated KHYG-1 cells. In each condition, an additional LRA was supplemented to the culture; prostratin (2 µM), JQ1 (1 µM), romidepsin (17.5 nM), or no LRA. J-Lat cells were cultured under these conditions for 22 h at 37°C.

For 1C10 cells, cells were exposed to latency-reversal agents PMA (50 ng/ml) in combination with ionomycin (1 μM), or prostratin (6 μM), or supernatants of KHYG-1 for 16 h.

### Flow cytometry

For the co-culture assay and the incubation with supernatant from NK cells experiment, J-Lat 10.6 cells were washed in ice-cold PBS and incubated with Near IR viability dye for 30 min at 4°C in the dark. The cells were then washed with ice-cold PBS and fixed with 2% paraformaldehyde for 20 min at 4°C in the dark. They were washed with ice-cold PBS one last time and finally resuspended in ice-cold PBS and stored at 4°C until they were analyzed. For 1C10 cells, cells were washed once with PBS, stained with Violet Dead Cell Stain and fixed in 2% formaldehyde for 15 min.

For the CCR5 expression experiment, J-Lat 10.6 cells were kept in five different conditions and TZM-bl cells were used as a positive control. On day one, a cell suspension of 1x10^6^ KHYG-1 cells/mL was stimulated with Z-VAD-FMK overnight at 37°C. On day two, J-Lat cells were resuspended to a concentration of 0.5x10^6^ cells/mL in (1): only RPMI 1640 medium (2), 100 U/mL IL-2, (3) Z-VAD-FMK, (4) 3 mL of supernatant of KHYG-1 cells or (5) 3 mL of supernatant of KHYG-1 cells that were stimulated with Z-VAD-FMK. All cells were incubated overnight at 37°C. On day three, cells were washed twice in FACS buffer (PBS with 1% BSA and 2 mM EDTA) and incubated with no antibody, BV421 isotype or CCR5-BV421 antibody diluted in FACS buffer with Near IR viability dye for 30 min at 4°C in the dark. The cells were then washed with FACS buffer and fixed with 2% paraformaldehyde for 20 min at 4°C in the dark. They were washed twice with ice-cold PBS and finally resuspended in ice-cold PBS and stored at 4°C until they were analyzed.

For staining of NK cell populations in PBMCs or enriched primary NK cell cultures, 0.5x10^6^ to 1.0x10^6^ cells were collected per staining. Cells were washed twice in ice-cold FACS buffer and incubated with CD3-PE, CD16-PerCP-Cy5.5, CD56-PE-Cy7 antibodies and Near IR viability dye for 30 min at 4°C in the dark. The cells were then washed with FACS buffer and fixed with 2% paraformaldehyde for 20 min at 4°C in the dark. They were washed twice with ice-cold PBS and finally resuspended in ice-cold PBS and stored at 4°C until they were analyzed.

All flow analysis was performed on BD FACSVerse (BD Biosciences), except for the analysis of 1C10 cells that was performed on a CytoFLEX S (Beckman Coulter, Pasadena, CA, USA).

### Luciferase assay

TZM-bl cells (2x10^4^ cells) were seeded in 96-wells plate one day prior to start of the experiment. Then, medium was removed, and supernatants of NK cells (200 µL) were added in triplicate. TZM-bl cells with only complete RPMI medium, RPMI with pan-caspase inhibitors (50 µM), RPMI with rhIL-2 (either 100 units/mL for experiments with KHYG-1 cells or 1000 units/mL for experiments with enriched primary NK cells), and RPMI with prostratin (6 µM) were used as controls. After one day of culture, supernatants were removed, washed once with PBS, and lysed in 50 µL Passive Lysis Buffer (Promega, Madison, WI, USA) for 30 min at 4°C. Finally, 20 µL lysates were transferred into a white 96-wells plate, 100 uL luciferase reagent (Promega) was added, and luminescence was measured on the Tecan Infinite^®^ 200 Pro microplate reader (Tecan Group Ltd, Männedorf, Switzerland). For each experiment fold change was calculated by dividing the relative luminescence units (RLU) of each condition by the RLU of TZM-bl cells cultured only with complete RPMI medium.

### Cytokine array

A cell suspension of 1x10^6^ KHYG-1 cells/mL was incubated with Z-VAD-FMK overnight at 37°C. The following day, 700 µL of supernatant was used to perform a Proteome Profiler™, Human Cytokine Array (#ARY005B, R&D Systems, Minneapolis, MN, USA) according to the instructions of the manufacturer. This array allows the determination of the relative expression levels of 36 human cytokines. Chemiluminescence was detected by ChemiDoc XRS+ (Bio-Rad, Hercules, CA, USA).

### Quantitative PCR

A cell suspension of 1x10^6^ KHYG-1 or primary NK cells/mL were stimulated with Z-VAD-FMK and incubated overnight at 37°C. Total RNA was extracted with Quick-RNA™ Miniprep Plus Kit (Zymo Research, Irvine, CA, USA) according to the instructions of the manufacturer. cDNA was synthesized using High-Capacity cDNA Reverse Transcription Kit (Applied Biosystems, Waltham, MA, USA), with RNasin^®^ Ribonuclease Inhibitor (Promega). qPCR reactions were done using Premix Ex Taq™ (Probe qPCR) (Takara Bio Inc., Kusatsu, Japan) with the primer/probe sets (#4331182) for CCL3 (assay ID Hs00234142_m1), CCL4 (Hs99999148_m1), CCL5 (Hs99999048_m1) and GAPDH (Hs02786624_g1) from ThermoFisher Scientific. The qPCR was performed on an ABI Fast 7500 system (Applied Biosystems) according to manufacturer’s protocol. Gene expression analysis was performed using the ΔΔCT method. RNA samples were stored at -20°C and cDNA samples at 4°C.

### Enrichment primary NK cells

PBMCs were isolated from buffy coats of anonymous blood donors using Ficoll density centrifugation (Ficoll^®^ Paque Plus, Sigma), aliquoted and stored in liquid nitrogen. A frozen aliquot of PBMCs was thawed and NK cells were negatively selected by incubating the sample in PBS containing 2% FBS with 1 mM EDTA and 50 µL/mL of Enrichment Cocktail (Stem Cell Technology, Vancouver, Canada) for 10 min at RT; followed by incubation with 100 µL/mL Magnetic Particles for 5 min at RT and the usage of a magnet to collect the supernatant containing the enriched NK cells. These cells were kept in AIM-V medium (Gibco) supplemented with 5% human AB serum (Sigma). The first day of culture, 1000 U/mL of rhIL-2 and 20 ng/mL of rhIL-21 was added. After the first day of culture, a daily dose of only 1000 U/mL of rhIL-2 was added. After 4 days of culture, enriched NK cells were treated with Z-VAD-FMK or DMSO for one day.

### Proximity ligation assay

J-Lat 1C10 cells were washed with PBS and allowed to adhere to Polylysine coated slides (VWR, Radnor, PA, USA) marked with a hydrophobic barrier using A-PAP pen (Histolab, Gothenburg, Sweden). PLA was performed according to the manufacturer’s protocol (Sigma) with a few modifications: PLA plus and minus probes were diluted 1:20, amplification buffer (5×) was used at 10×. All washes were performed in PBS. Antibodies (1:1,000) were used against Tat (Abcam, Cambridge, United Kingdom), FLAG M2 (Sigma). Before DAPI staining and mounting with Duolink in Situ mounting media with DAPI (Sigma), FITC-conjugated anti-GFP (Abcam) was applied (1:500) for 1 h at ambient temperature protected from light. Slides were sealed with nail polish and stored at 4°C overnight before imaging. Slides were imaged using a Pannoramic Midi II slide scanner (3DHistech, Budapest, Hungary) and images were exported using the CaseViewer application. Images were analyzed with ImageJ (version 2.0.0-rc-69/1.52) and macros developed in house. Each RGB image was separated into separate channels. Based on the blue (DAPI) channel, nuclei were identified. In the red channel, spots (“maxima”) were detected with prominence thresholds 6–48.

### Data analysis

Data was analyzed and plotted using GraphPad Prism v8.4.2 or v9.0.0 (San Diego, CA, USA). Statistical analysis using ANOVA was performed using GraphPad Prism.

## Results

### NK cells induce and increase HIV-1 reactivation after treatment with pan-caspase inhibitor Z-VAD-FMK

**Figure 1:**
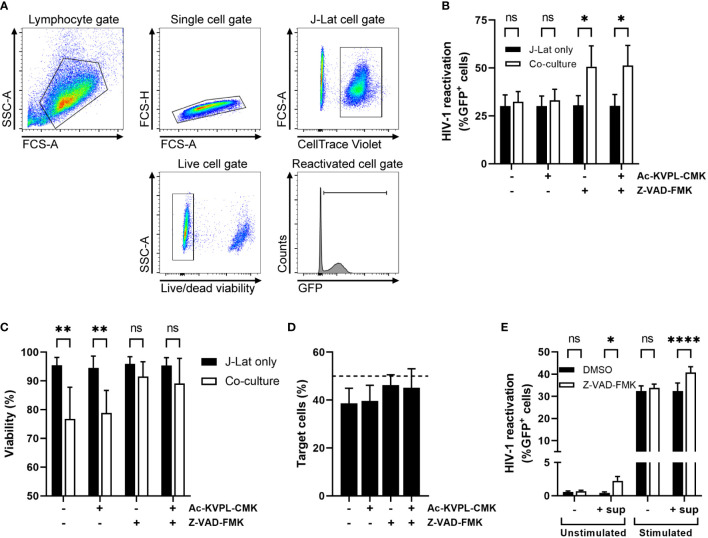
Z-VAD-FMK-treated NK cells induce HIV-1 reactivation from latency. **(A)** Gating strategy in our co-culture cell model for flow cytometry analysis. **(B)** HIV-1 latently infected J-Lat cells stimulated with LRA prostratin were cultured in the presence of GrM-inhibitor Ac-KVPL-CMK or pan-caspase inhibitor Z-VAD-FMK either with or without KHYG-1 NK cells. HIV-1 reactivations as measured by GFP-positivity was assessed by flow cytometry. **(C)** Cell viability of J-Lat cells was assessed by near infrared viability dye staining and flow cytometry. **(D)** The percentage of CellTrace Violet-positive J-Lat cells in co-cultures was measured by flow cytometry. **(E)** Unstimulated or prostratin-stimulated J-Lat cells were incubated with supernatants of Z-VAD-FMK-treated NK cells. HIV-1 reactivations as measured by GFP-positivity was assessed by flow cytometry. Data points are plotted as mean ± SD from three individual experiments. (ns, non-significant; *p < 0.05, **p < 0.01, ****p < 0.0001; ANOVA).

Since we were interested in whether NK cells could inhibit HIV-1 replication in a GrM-dependent manner, we performed a co-culture experiment using the HIV-1 latently infected cell line J-Lat 10.6 as target cells and the NK cell lymphoma cell line KHYG-1, which has high expression levels of GrM but not granzyme B, as effector cells. J-Lat cells contain an integrated copy of HIV-1 with GFP in place of Nef, an accessory gene. Thus, HIV-1 reactivation and replication can be monitored by GFP expression. We also labelled J-Lat cells with CellTrace Violet to discriminate them from KHYG-1 cells in flow cytometry analysis (**Figure 1A**). We briefly reactivated J-Lat cells with the LRA prostratin and then co-cultured them with KHYG-1 cells in an 1:1 effector:target (E:T) ratio for 1 day. GrM inhibitor Ac-KVPL-CMK was added to specifically inhibit the proteolytic activity of GrM. We included the pan-caspase inhibitor Z-VAD-FMK in the co-cultures to limit bias of target cell death. The GrM inhibitor had no effect on the reduction in percentage of GFP-positive J-Lat cells when co-cultured with KHYG-1 cells (**Figure 1B**). The presence of Z-VAD-FMK prevented cell death of J-Lat cells induced by KHYG-1 cells (**Figure 1C**) and maintained the E:T ratio of 1:1 throughout the experiment (**Figure 1D**). The percentage of GFP-positive J-Lat cells was increased when co-cultured with KHYG-1 and in the presence of Z-VAD-FMK (**Figure 1B**). This effect only occurred in co-culture with KHYG-1 cells, but not when J-Lat cells were cultured alone with Z-VAD-FMK.

Next, we assessed whether cell-to-cell contact was required for Z-VAD-FMK-treated KHYG-1 cells to induce HIV-1 reactivation. We incubated KHYG-1 cells with Z-VAD-FMK for one day, collected the supernatant, and cultured either unstimulated or prostratin-stimulated J-Lat cells with the supernatants. Supernatants of Z-VAD-FMK-treated KHYG-1 cells induced and increased the percentage of GFP-positive J-Lat cells (**Figure 1E**). Thus, the pan-caspase inhibitor Z-VAD-FMK appeared to affect the KHYG-1 cells to secrete a biological product that subsequently induces or increases HIV-1 reactivation within J-Lat cells.

### Synergistic effects on HIV-1 reactivation of pan-caspase inhibitor-treated NK cells with other LRAs

**Figure 2:**
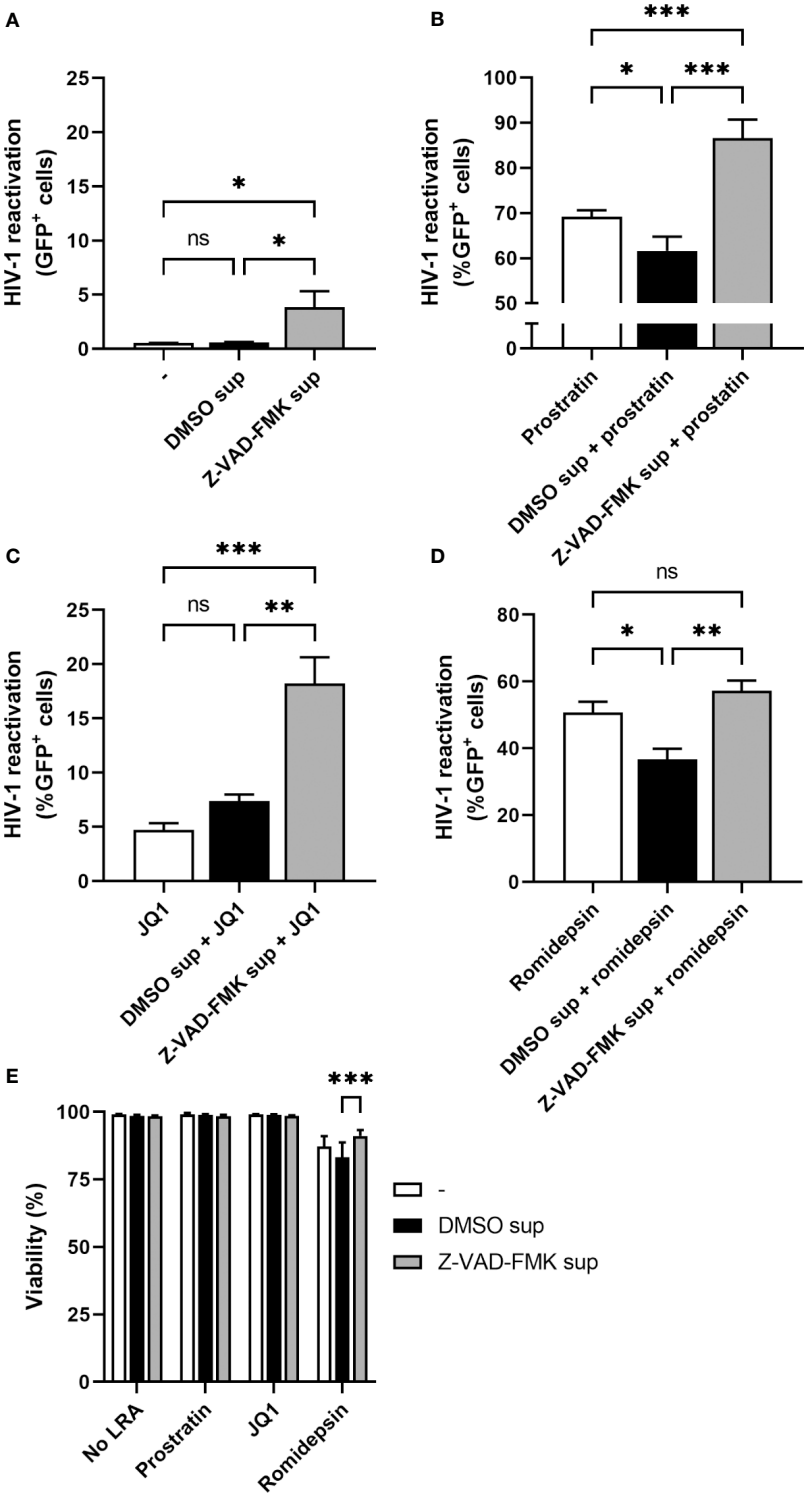
Supernatants of pan-caspase inhibitor-treated NK cells work in synergy with other LRAs. KHYG-1 cells were incubated with Z-VAD-FMK or only DMSO. After 24 h, supernatants were collected. Then, J-Lat cells were cultured in normal media or in the collected supernatants without any additional LRAs **(A)**, or with added prostratin (2 µM) **(B)**, JQ1 (1 µM) **(C)**, or romidepsin (17.5 nM) **(D)** for 22 h. HIV-1 reactivations as measured by GFP-positivity was assessed by flow cytometry. **(E)** Cell viability of J-Lat cells was assessed by near infrared viability dye staining and flow cytometry. Data points are plotted as mean ± SD from three individual experiments. (ns, non-significant; *p < 0.05, **p < 0.01, ***p < 0.001; ANOVA).

Since the supernatant of Z-VAD-FMK-treated KHYG-1 cells only weakly induced HIV-1 reactivation in J-Lat cells, we tested whether there could be synergy in HIV-1 reactivation with some other LRAs. For this, we cultured J-Lat cells in normal medium or in collected KHYG-1 supernatants. In each of these conditions we either added prostratin (protein kinase C agonist), JQ1 (BET bromodomain inhibitor), romidepsin (histone deacetylase inhibitor), or no additional LRA and measured the percentage of GFP-positive cells by flow cytometry after one day of culture. Again, supernatants of Z-VAD-FMK-treated KHYG-1 weakly induced HIV-1 reactivation with approximately 3.9% GFP-positive J-Lat cells without any additional LRA (**Figure 2A**). We observed an increase in prostratin-induced reactivation from 69.2% to 86.6% and JQ1-induced reactivation from 4.7% to 18.2% when J-Lat cells were cultured in supernatants of Z-VAD-FMK-treated KHYG-1 cells (**Figures 2B, C**). These increases in GFP-positivity were clearly higher than the added percentages of GFP-positivity induced by the supernatant of Z-VAD-FMK-treated KHYG-1 or the individual LRA alone, indicating a synergistic effect. For romidepsin, there was no significant increase in the percentage of GFP-positive J-Lat when cultured in supernatants of Z-VAD-FMK-treated KHYG-1 cells (57.2%) compared to romidepsin-treated J-Lat cells (50.7%) (**Figure 2D**), although it was significantly increased compared to DMSO-treated supernatants (36.7%). Except for romidepsin treatment, viability was equally high in all conditions and thus did not affect the percentages of GFP-positivity cells (**Figure 2E**). Therefore, treatment of KHYG-1 cells with Z-VAD-FMK can work in synergy with some other LRAs to induce a stronger HIV-1 reactivation.

### Pan-caspase inhibitor-treated NK cells induce HIV-1 promoter activity through a secreted factor

**Figure 3:**
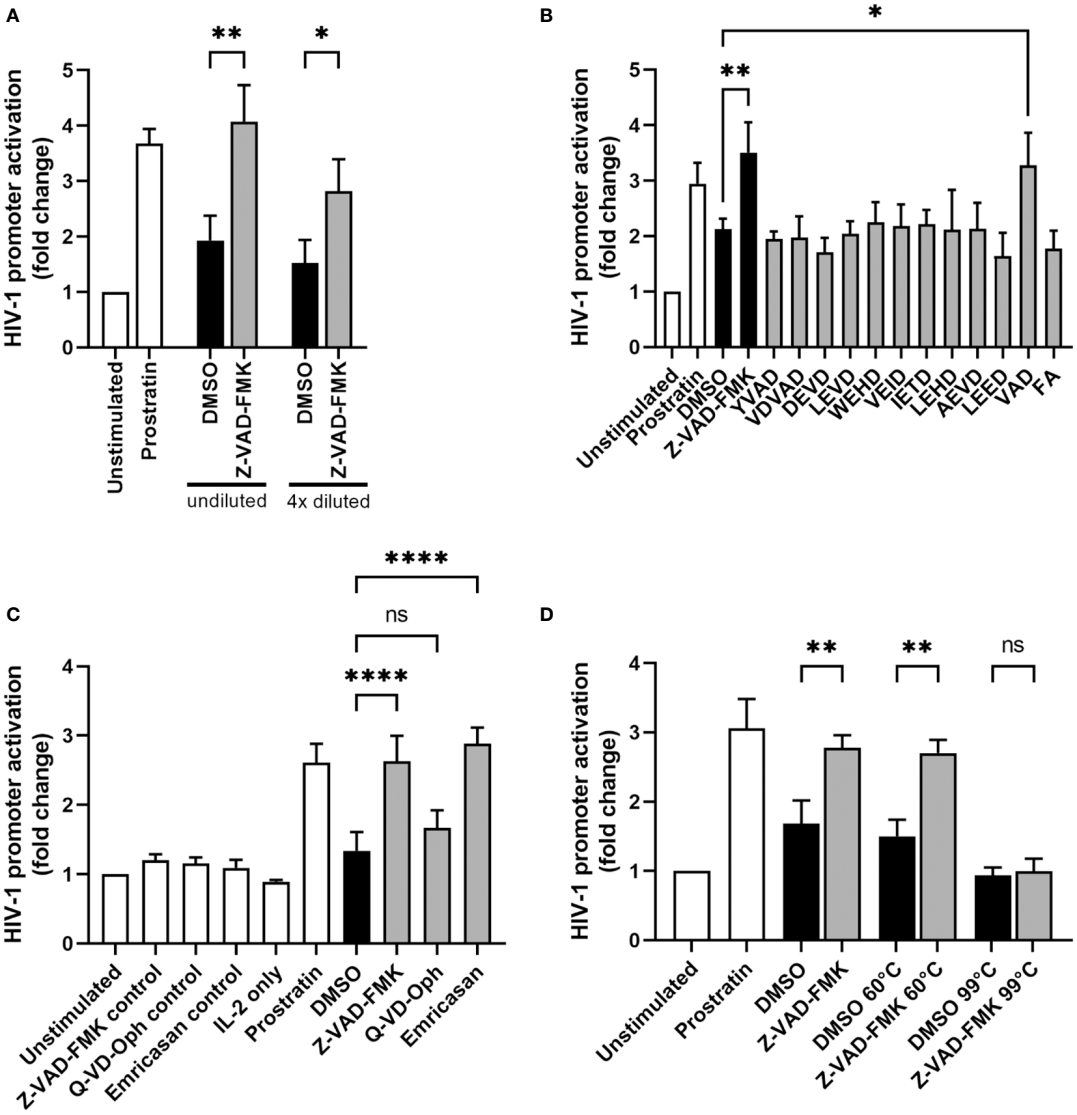
Supernatants of pan-caspase inhibitor-treated NK cells induce HIV-1 promoter activity. **(A)** KHYG-1 cells were incubated with Z-VAD-FMK or only DMSO. After 24 h, supernatants were collected and added to reporter cell line TZM-bl. Activation of the HIV-1 LTR promoter was assessed by subjecting these cells to a luciferase assay (n=3). Luminescence values of unstimulated cells was set to 1. TZM-bl cells incubated with LRA prostratin served as positive control. **(B)** KHYG-1 cells were incubated with various specific caspase inhibitors and their supernatants were analyzed as in (A) (n=3). All individual caspase inhibitors had the same chemical structure as Z-VAD-FMK, except for the varying peptide sequence. **(C)** KHYG-1 cells were incubated with various pan-caspase inhibitors and their supernatants were analyzed as in (A) (n=4). TZM-bl incubated with IL-2 or pan-caspase inhibitors were used as controls. **(D)** KHYG-1 cells were incubated with Z-VAD-FMK and their supernatants were heat-inactivated at either 60°C, 99°C or left untreated before adding to TZM-bl cells and performing a luciferase assay (n=2). Data points are plotted as mean ± SD. (ns, non-significant; *p < 0.05, **p < 0.01, ****p < 0.0001; ANOVA).

Since HIV-1 reactivation starts with the activation of the HIV-1 long terminal repeat (LTR) promoter, we examined whether the supernatants of Z-VAD-FMK-treated KHYG-1 cells can induce HIV-1 promoter activation. For this we used TZM-bl cells, which have an integrated copy of the luciferase gene under the HIV-1 LTR promoter. First, KHYG-1 cells were incubated with or without pan-caspase inhibitor Z-VAD-FMK for 1 day. Then, we collected the supernatants and cultured the TZM-bl cells in these supernatants for 1 day before we performed a luciferase assay. Prostratin was used as a positive control and showed increased HIV-1 promoter activity (**Figure 3A**), as expected. Supernatant of Z-VAD-FMK-treated KHYG-1 induced significantly higher HIV-1 promoter activity, compared to DMSO-treated supernatants (**Figure 3A**). HIV-1 promoter activity decreased when we diluted the supernatants. Altogether, this indicates that a secreted factor by KHYG-1 cells after treatment with the pan-caspase inhibitor Z-VAD-FMK can induce activation of the HIV-1 LTR promoter.

Since TZM-bl cells do not express Tat, we used J-Lat 1C10 cells as another cell model to examine whether the reactivation was Tat-driven or Tat-independent (20). This cell line allows detection of transactivation response element (TAR)-bound Tat in proximity to an LTR-bound zinc finger protein through a proximity ligation assay. Although the parental cell line 5A8 is known to be less responsive to some common LRAs (21), we did observe a significant, albeit low, increase in GFP positivity of the 1C10 cells when cultured with supernatants of Z-VAD-FMK-treated KHYG-1 cells compared to supernatants of control KHYG-1 cells (**Supplementary Figure 1A**). However, no increase in LTR-bound Tat was detected in the 1C10 cells that were incubated with supernatants of Z-VAD-FMK-treated KHYG-1 cells (**Supplementary Figure 1B**). This indicates that the observed reactivation occurs before Tat binding to the TAR region of HIV-1 RNA.

Since Z-VAD-FMK inhibits a wide range of caspases, we tested more specific caspase inhibitors to figure out which inhibited caspase within the KHYG-1 cells was responsible for the NK-cell mediated induction of HIV-1 promoter activity. We incubated the KHYG-1 cells with ten specific caspase inhibitors as well as with a positive control inhibitor (VAD) that is identical to the Z-VAD-FMK used in all our other experiments and a negative control inhibitor (FA). All inhibitors contain the N-terminal benzyloxycarbonyl (Z-) group and C-terminal fluoromethylketone (FMK) inhibitor group. After 24 h, supernatants were collected and added to TZM-bl cells. However, none of the specific caspase inhibitors alone could increase HIV-1 promoter activity (**Figure 3B**).

Further, we examined whether two other pan-caspase inhibitors, Q-VD-Oph and emricasan, could cause KHYG-1 cells to activate the HIV-1 promoter. Q-VD-Oph did not increase the ability of KHYG-1 supernatants to induce HIV-1 promoter activity, whereas emricasan activated the HIV-1 promoter similar to Z-VAD-FMK (**Figure 3C**). Heat-inactivation of the supernatants at 60°C before adding them to the TZM-bl cells, did not alter the capacity of supernatants of Z-VAD-FMK-treated KHYG-1 cells to induce HIV-1 promoter activity (**Figure 3D**). Heat-inactivation at 99°C abolished HIV-1 promoter activation by the supernatants.

**Figure 4:**
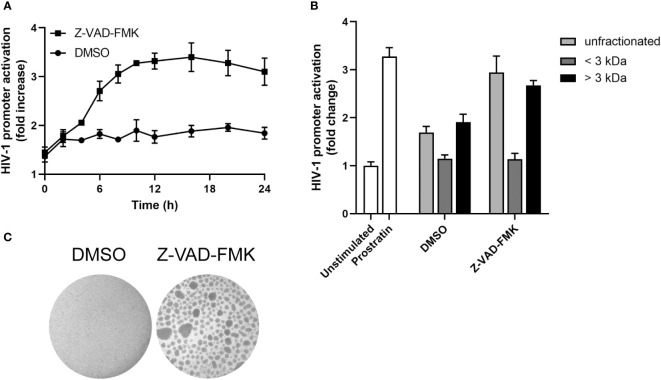
Secreted factor of pan-caspase inhibitor-treated NK cells is most likely a cytokine. **(A)** KHYG-1 cells were incubated with Z-VAD-FMK or only DMSO for various time points after which supernatants were collected and added to TZM-bl cells. Activation of the HIV-1 LTR promoter was assessed by subjecting these cells to a luciferase assay (n=3). Luminescence values of unstimulated cells was set to 1. **(B)** Supernatants of KHYG-1 cells were collected and fractionated using columns with a 3 kDa cut-off. Unfractionated or fractionated supernatants were added to TZM-bl cells and analyzed as in (A) (n=3). **(C)** Light microscopy pictures of KHYG-1 cells treated with Z-VAD-FMK or DMSO for 24 h. For all graphs, data points are plotted as mean ± SD.

Next, we wanted to assess the kinetics of Z-VAD-FMK induced HIV-1 promoter activation. In a time course experiment, we observed an increase in HIV-1 promoter activity with supernatants of KHYG-1 treated with Z-VAD-FMK for 8 h, followed by a plateau (**Figure 4A**). We also separated the supernatant of Z-VAD-FMK-treated KHYG-1 cells into two fractions based on size with a cut-off of 3 kDa. The fraction below 3 kDa showed no activity, whereas the fraction above 3 kDa showed the same activity as unfractionated supernatants (**Figure 4B**). Finally, we observed that Z-VAD-FMK-treated KHYG-1 cells were forming clumps (**Figure 4C**), indicating a proliferating or activated phenotype. However, since there was no increase in proliferation (**Figure 1D**, and data not shown), it likely represented activation of the cells. Altogether, these data suggest that Z-VAD-FMK-treated KHYG-1 cells induce HIV-1 promoter activity in target cells most likely through a secreted protein.

### Pan-caspase inhibitor-treated NK cells secrete increased levels of HIV-1 suppressor cytokines

**Figure 5:**
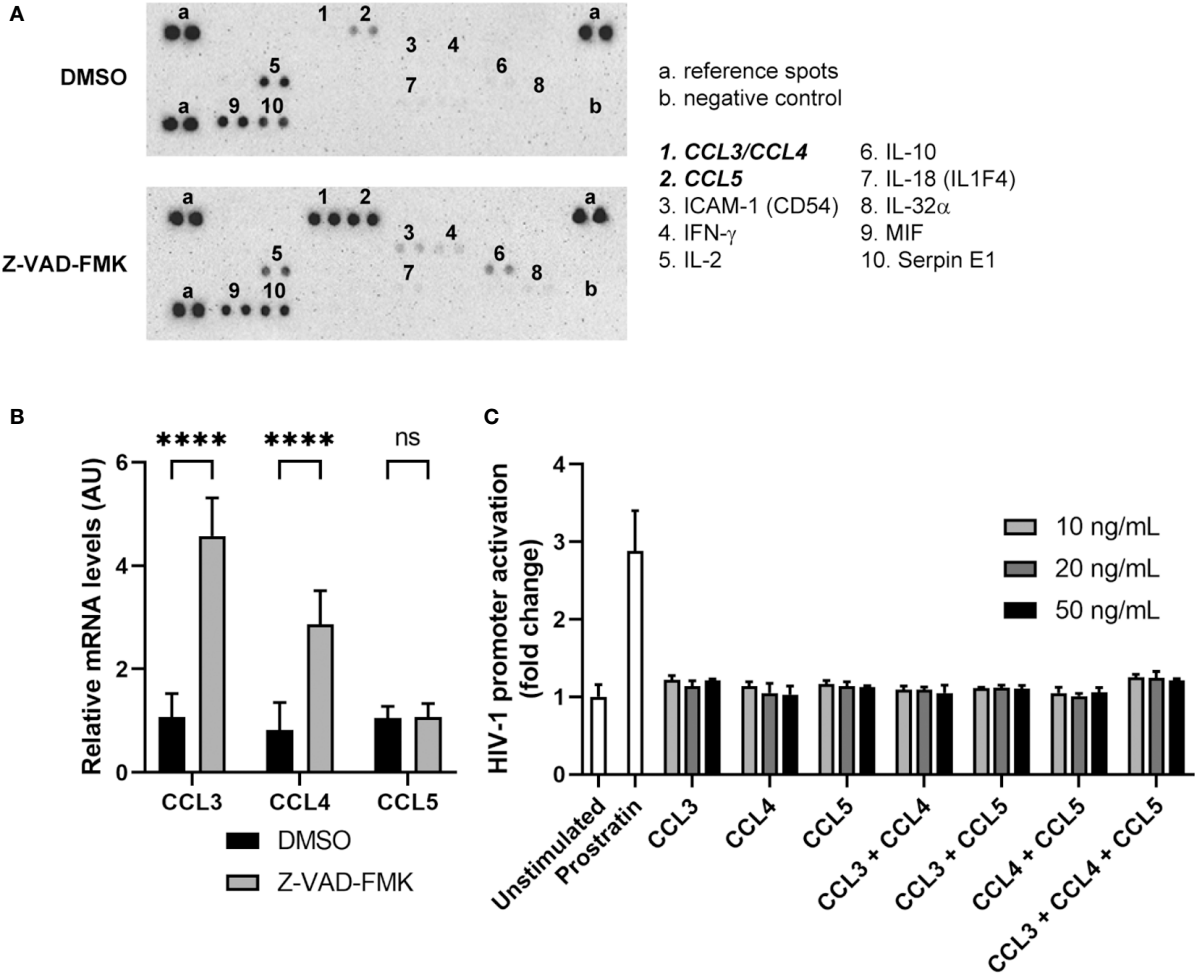
Pan-caspase inhibitor-treated NK cells secrete increased levels of HIV-1 suppressor cytokines. **(A)** KHYG-1 cells were incubated with Z-VAD-FMK or only DMSO. After 24 h, supernatants were collected and subjected to a cytokine array. **(B)** RNA from Z-VAD-FMK-treated or DMSO-treated (control) KHYG-1 cells was isolated and mRNA levels of CCL3, CCL4, CCL5, and GAPDH was measured by qPCR. GAPDH was used to normalize for input, and the average mRNA level for DMSO (n=6) was set to 1. **(C)** TZM-bl cells were incubated with various concentrations of CCL3, CCL4, CCL5, or combinations of these three chemokines (n=3). After 24 h, activation of the HIV-1 promoter was measured by luciferase assay. For all graphs, data points are plotted as mean ± SD. (ns, non-significant; ****p < 0.0001; ANOVA).

Since NK cells can exert their functions through the secretion of a variety of cytokines, we examined whether there was a difference in the cytokine protein profile of Z-VAD-FMK-treated KHYG-1 cells that could inform how HIV-1 reactivation occurred in the target cells. We collected supernatants of control or Z-VAD-FMK-treated KHYG-1 cells and subjected the supernatants to a cytokine array that can detect protein levels of 36 different cytokines (**Figure 5A**; **Supplementary Figure 2**). Since KHYG-1 requires recombinant human IL-2 for growth in culture, we detected equal amounts of IL-2 in both supernatants. MIF and SerpinE1 were also identified in equal levels in the supernatants of control and Z-VAD-FMK-treated KHYG-1. However, protein levels of the cytokines MIP1α (CCL3), MIP1β (CCL4) and RANTES (CCL5) increased in the supernatants of Z-VAD-FMK-treated KHYG-1 cells. These three cytokines are ligands for the HIV-1 entry co-receptor CCR5 and act as HIV-1 suppressors by blocking entry of HIV-1 into the target cells (22–24). Then, we examined the effect of Z-VAD-FMK on the mRNA levels of CCL3, CCL4, and CCL5 within KHYG-1 cells by qPCR. Both CCL3 and CCL4 mRNA levels were increased after Z-VAD-FMK treatment, whereas CCL5 mRNA levels were not affected by the treatment (**Figure 5B**).

To assess whether the cytokines CCL3, CCL4, and CCL5 were responsible for the HIV-1 reactivation, we incubated TZM-bl cells with different physiological concentrations of each of the cytokines. Neither individually nor in combinations did these cytokines induce HIV-1 promoter activity (**Figure 5C**). Also, at a non-physiologically high dose we could not detect HIV-1 promoter activation by these chemokines (**Supplementary Figure 3A**). Although the J-Lat cells used in this study did not express CCR5 as measured by flow cytometry (**Supplementary Figure 3B**), we did also examine whether these cytokines could induce HIV-1 reactivation in these cells in a CCR5-independent manner. No increase in GFP expression was observed after J-Lat cells were incubated with any or combinations of the three cytokines (**Supplementary Figure 3C**). Altogether, Z-VAD-FMK induced secretion of CCL3, CCL4, and CCL5 by KHYG-1 cells, but these HIV-1 suppressor cytokines were not responsible for the reactivation of HIV-1.

### Primary NK cells can induce HIV-1 promoter activity after pan-caspase treatment

**Figure 6:**
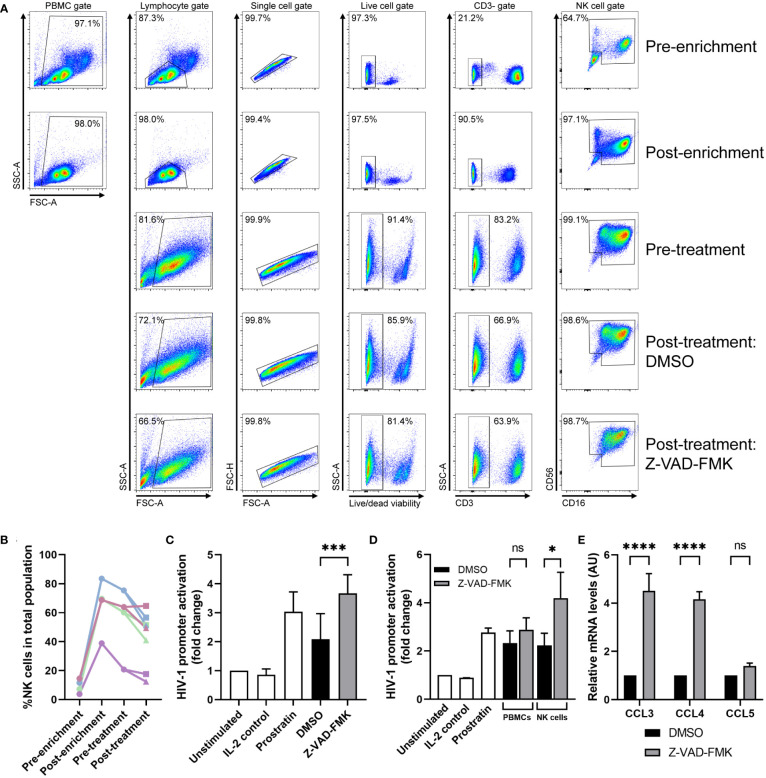
Primary NK cells can induce HIV-1 promoter activity after pan-caspase treatment. **(A)** Flow cytometry analysis on semi-enriched primary NK cells of one donor at different stages of the experiment; before semi-enrichment, after semi-enrichment, after 4 days of culture, and after 24 h treatment with DMSO or Z-VAD-FMK. **(B)** The percentage of all NK cells within the total cell population was plotted for each donor at each stage of the experiment. The values for post-treatment are divided between DMSO treatment (■) and Z-VAD-FMK treatment (▲). **(C)** Semi-enriched primary NK cells (from 8 donors) were cultured with Z-VAD-FMK or without (DMSO) for 24 h after which supernatants were used on reporter cells TZM-bl that were subjected to luciferase assay. **(D)** Semi-enriched primary NK cells (from 3 donors) or IL-2 activated whole PBMCs (from the same 3 donors) were cultured with Z-VAD-FMK or without (DMSO) for 24 h after which supernatants were used on reporter cells TZM-bl that were subjected to luciferase assay. **(E)** RNA was extracted from semi-enriched primary NK cells (4 donors) cultured with or without Z-VAD-FMK and mRNA expression of CCL3, CCL4, CCL5, and GAPDH (for normalization) was measured by qPCR. For all graphs, data points are plotted as mean ± SD. (ns, non-significant; *p < 0.05, ***p < 0.001, ****p < 0.0001; ANOVA).

To study the effect of pan-caspase inhibitors under more physiological conditions, we assessed whether Z-VAD-FMK can modulate primary NK cells to secrete HIV-1 latency reversal factors. We purified primary NK cells from primary PBMCs and after 4 days of *ex vivo* culture, we incubated the cells with Z-VAD-FMK, or DMSO as control. Although we obtained highly purified primary NK cell cultures following enrichment protocols, the culture showed low viability and proliferation in the following days as well as inconsistent results on HIV-1 promoter activation (data not shown). Since primary NK cells are highly dependent on cytokines and stimulatory signals from other (feeder) cells (25), we performed a semi-enrichment that yielded less pure primary NK cell cultures but could be consistently cultured *ex vivo* for several days. Following NK cell dynamics of CD3^-^CD56^dim^CD16^+^ and CD3^-^CD56^bright^CD16^-^ NK cells together in cultures from four donors showed high variation in the percentage of NK cells within PBMCs at the start of the semi-enrichment (3%-16%) and during the experiment (**Figure 6A**; **Supplementary Figures 4–6**). Since CD3^+^ T cells proliferate at a higher rate in response to IL-2 in the first days of ex vivo culture compared to NK cells, there is a minor drop in NK cell frequency. The one-day treatment of Z-VAD-FMK decreased the percentage of NK cells within the culture population (**Figure 6B**). This is most likely due to increased survival of mainly other cells in the culture where cell death was inhibited by Z-VAD-FMK. Analysis of semi-enriched *ex vivo* NK cell cultures from eight anonymous donors showed that supernatants of Z-VAD-FMK-treated cultures increased activation of the HIV-1 promoter compared to the control cultures (**Figure 6C**). This activation appears to be mediated by NK cells, since supernatant of Z-VAD-FMK-treated whole PBMCs show very low increased HIV-1 promoter activation (**Figure 6D**). Finally, we extracted mRNA from all cells in the Z-VAD-FMK-treated or control *ex vivo* semi-enriched primary NK cell cultures. Similar to the KHYG-1 cell line, we observed an increase in CCL3 and CCL4 mRNA expression in the Z-VAD-FMK-treated cells (**Figure 6E**). This data indicates that treatment of primary NK cells with a pan-caspase inhibitor induces the upregulation of CCL3 and CCL4 mRNA levels as well as secretion of a latency reversal factor.

## Discussion

**Figure 7:**
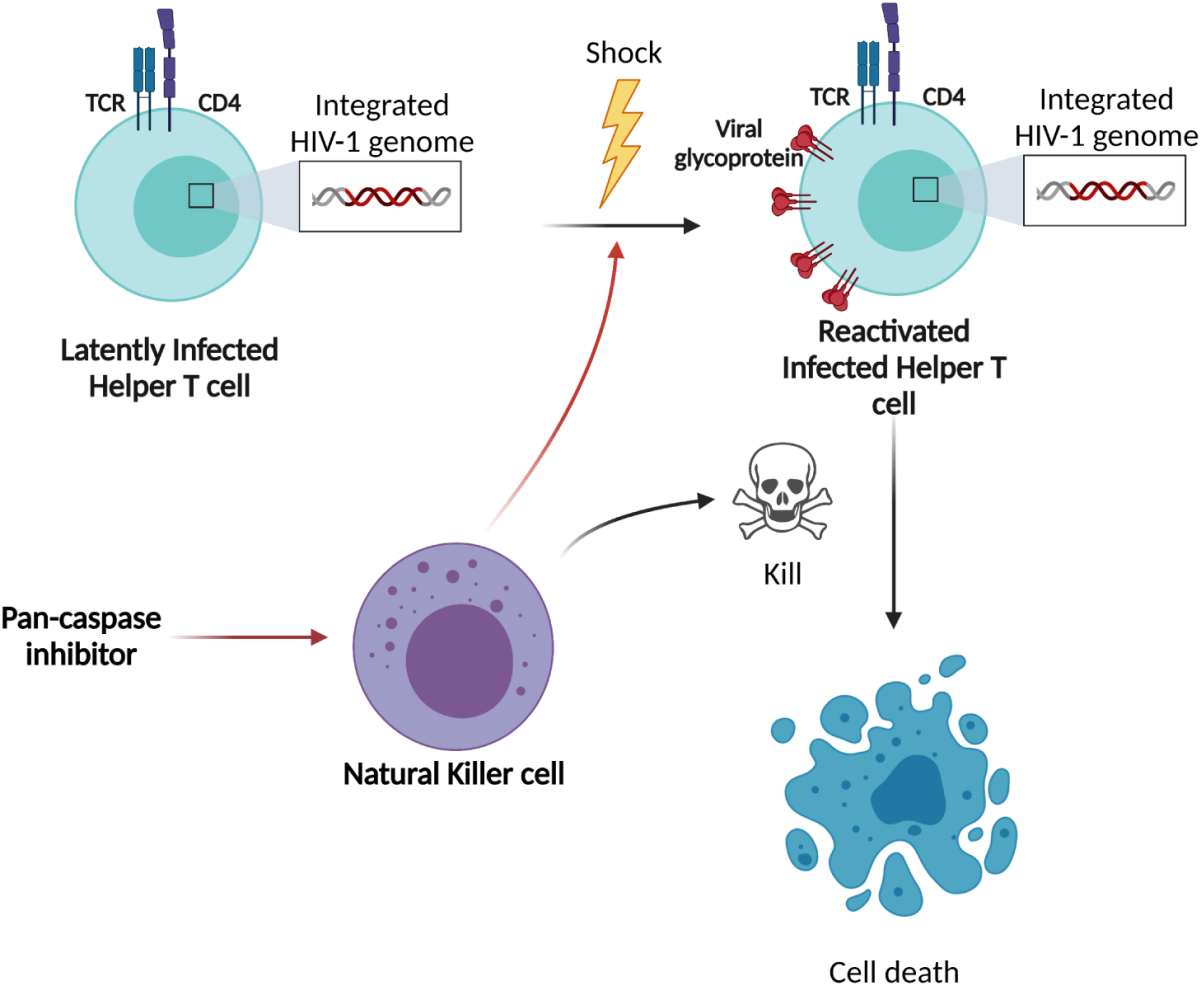
Schematic overview of shock-and-kill strategy and our findings. The original shock-and-kill strategies use LRAs to directly stimulate the HIV-1 latently infected cells. After latency reversal, these HIV-1 reactivated cells are eliminated by cytotoxic lymphocytes, such as NK cells. Based on our data, our proposed shock-and-kill strategy aims to stimulate NK cells with pan-caspase inhibitors resulting in the release of an LRA from the NK cells to provide the “shock” towards HIV-1 latently infected cells, followed by the “kill” of the reactivated cells by the same NK cells.

Latently HIV-1 infected cells are major barriers to an HIV-1 cure and these latently infected cells are impossible to distinguish from uninfected cells (1, 26). To break HIV-1 latency, epigenetic modulation of the provirus or activation of specific transcription factors that bind the HIV-1 LTR promoter must occur (7). Here, we observed for the first time that NK cells can acquire a “shock” ability when stimulated with pan-caspase inhibitors (**Figure 7**). In various experiments, we showed that pan-caspase inhibitors modulate NK cell activity resulting in the release of an LRA. If the NK-cells can be redirected through external stimuli, a potential advantage is that the approach will utilize both the “shock” ability of the LRA induced from the NK cells and the “kill” ability of the NK cells.

The intensity of reactivation by the LRA released by pan-caspase inhibitor treated NK cells varied in our cell models. Since we observed activation of the LTR in TZM-bl cells that lack the expression of Tat, we aimed to assess whether the NK cell induced reactivation was Tat-dependent or -independent in the 1C10 cell model. Although we observed the latter, the frequency of reactivation in these 1C10 cells was very low and this could have affected the detection of LTR-bound Tat. Indeed, the response to known LRAs can greatly differ among different HIV-1 latency models (21, 27). In case of the J-Lat cell lines, the 1C10 cell line originates from 5A8 cells that in turn, and unlike 10.6, is derived from selection through anti-CD3/anti-CD28 responsiveness (28, 29). Also, both cell lines have a different HIV-1 DNA integration site within the host chromatin (28, 30). These differences could cause variations in sensitivities to LRA signalling pathways. Since there is heterogeneity of the HIV-1 latent reservoir *in vivo*, various cell models are required to study the effects of LRAs, including our LRA secreted by pan-caspase-inhibitor treated NK cells. A strategy to induce HIV-1 reactivation in more latently infected cells is to combine multiple LRAs. Indeed, whereas the supernatant of Z-VAD-FMK-treated KHYG-1 cells was only able to weakly induce HIV-1 reactivation, there was a synergistic effect when combined with other LRAs, such as prostratin or JQ1.

Based on the kinetics of the activity within stimulated NK cells, the LRA is above 3 kDa in size, and because NK cells are cytokine-producing immune cells, this LRA could potentially be a cytokine. The observation that HIV-1 reactivation in J-Lat cells incubated with supernatants of pan-caspase inhibitor treated NK cells was not as strong as in the co-culture experiments could indicate that cell-to-cell contact contributes to inducing reactivation or it could be because these cells tend to be close together when they are co-cultured. Therefore, a small amount of the LRA is enough to induce reactivation in HIV-1 latently infected cells co-cultured with NK cells, while it might be too diluted when HIV-1 latently infected cells are cultured only with the supernatant.

Since we speculated that the LRA could be a cytokine, we examined differences in the levels of secreted cytokines of NK cells after treatment with pan-caspase inhibitors. Although TNF, which is a known LRA usually released by activated NK cells after target cell recognition (27, 31–34), was undetectable in the supernatant of pan-caspase inhibitor-treated NK cells, we observed increased levels of CCL3, CCL4, and CCL5. On mRNA level, only CCL3 and CCL4, but not CCL5, were upregulated in pan-caspase inhibitor-treated NK cells. To our knowledge, the production of CCL3, CCL4 and CCL5 in human NK cells after stimulation with a pan-caspase inhibitor has not been studied. Possibly the pan-caspase inhibitor treatment induces release of pre-stored CCL5 or upregulation in translation, whereas the increased release of CCL3 and CCL4 is mediated through changes on a transcriptional level.

Despite having different immunological functions, the three cytokines are CCR5 ligands (22). CCR5 is the most commonly used co-receptor by HIV-1 to enter cells and, for this reason, these three cytokines normally act as HIV-1 suppressors by blocking viral entry into the host cell (23, 24). In the context of latent reservoirs, however, the HIV-1 genome is already integrated in the host cell genome, meaning the cytokines would not be inhibiting infection in our model. A recent study has shown that maraviroc, a CCR5 antagonist used for HIV-1 treatment, can act as a latency reversal agent (35), which reinforced our idea that CCL3, CCL4 and CCL5 could be inducing HIV-1 reactivation. Since these cytokines are CCR5 ligands and there is some controversy in literature regarding the J-Lat parental cell line Jurkat expressing cell surface CCR5 (36–40), we analyzed the expression on our J-Lat cells in several conditions that resembled the cell culture environment in our other experiments. CCR5 expression was not detected in those conditions. Nonetheless, we analyzed whether CCL3, CCL4 and CCL5 increased HIV-1 promoter activity or induced latency reversal, since they could be acting through another receptor and, besides that, physiologically latently infected cells express CCR5. No increase in HIV-1 reactivation was observed in CCR5-deficient J-Lat cells nor HIV-1 promoter activation in CCR5-expressing TZM-bl cells with any of the cytokines or the combinations. Therefore, although these cytokines are being upregulated, they are not responsible for the increase in HIV-1 reactivation in our models. However, their presence could provide additional HIV-1 suppression in the microenvironment of reactivated cells.

Usually, activation of NK cells is mediated by a balance of inhibitory and stimulatory cell surface receptors on both target cells and NK cells. This activation results in the release of various cytokines and chemokines as well as executing their cytotoxic activities (32, 34). Although we observed elevated levels of the chemokines CCL3, CCL4, and CCL5 and minor release in IL-10 and IFN-γ, we did not detect secretion of TNF, IP-10 or IL-8. This indicates that the pan-caspase inhibitors only partially activate the NK cells. This could be of clinical importance, because therapeutic activation of NK cells using pan-caspase inhibitors would limit iatrogenic systemic immune hyperactivation and the risk of inducing a cytokine storm (41). However, it would be interesting to further study to what extent the pan-caspase inhibitors activate the NK cells. Additionally, it would be interesting to examine the cytotoxic potential of the NK cells towards reactivated infected cells after the pan-caspase inhibitor treatment in way that the NK cells could execute both the “shock” and “kill” steps.

Caspases are produced as inactive pro-caspases and become activated after their specific stimuli. Although inflammatory caspases (caspases 1, 4, and 5) are known to regulate inflammation, initiator caspases (caspases 2, 8, 9, and 10) and executioner/effector caspases (caspases 3, 6, and 7) regulate apoptosis (42), which is why we initially used the pan-caspase inhibitor to block cell death. However, these caspases can also play underappreciated non-canonical roles. For example, apoptotic caspases can also have roles in a range of neuronal cell behaviours and even in antiapoptotic mechanisms (43, 44). Moreover, caspase 8, which is essential for death receptor-induced apoptosis, has also been shown to have a role in the homeostatic control in the adult immune system (45). Active caspase 8 is also required for activation-induced proliferation of T lymphocytes (46). More importantly, activate caspase 8 has been shown to be required for IL-2-induced proliferation of NK cells as well as release of IFN-γ and TNF from activated NK cells that can be inhibited by Z-VAD-FMK (47). Altogether, this shows that caspases have non-canonical roles and can potentially have non-described roles in several cellular mechanisms. Our data contributes to this emerging concept of non-canonical roles for caspases. Since we show that pan-caspase inhibitors affect NK cell function in inactive NK cells and others have shown effects in activated NK cells (47), it could be that some caspases are active during different phases of NK cells, *i.e.*, inactive resting state and activate state, and thereby regulating balanced NK cell effector functions. In future studies it would be of great interest to get a better understanding to what extent caspases could negatively regulate NK cell activation pathways in resting inactive NK cells and how we could potentially modulate NK cell activation in other biological or pathological contexts.

As of yet, we have not elucidated the identity of the LRA secreted by pan-caspase inhibitor-treated NK cells. For this, a proteomic approach is required to assess the full changes in the NK cell secretome after pan-caspase inhibitor treatment followed by validation in HIV-1 reactivation cell culture assays. Nonetheless, our novel finding that the pan-caspase inhibitors have the potential to modulate the activities of NK cells to produce an LRA resulting in reactivation of HIV-1 in latently infected cells provides for a unique opportunity to design a new combination strategy for the cure of HIV-1. In another study, a pan-caspase inhibitor was shown to inhibit death of T cells from SIV-infected rhesus macaques and was suggested to represent an adjunctive therapeutic agent to control HIV infection and delaying disease progression to AIDS (48). Although Z-VAD-FMK itself will not be useful as drug treatment, the pan-caspase inhibitor drug emricasan is currently in clinical trials for treatment of liver diseases (49–55) and could be repurposed without lengthy preclinical development. However, since the main function of pan-caspase inhibitors is inhibiting all caspases and consequently inhibiting caspase-induced cell death, we also inhibit cytotoxic lymphocyte-mediated target cell death of the HIV-1 reactivated cells. This could be circumvented by specifically targeting the NK cells for (pan-)caspase inhibitor drug delivery. Alternatively, we must further study how pan-caspase inhibitors modulate NK cells and which inhibiting caspases are responsible for the acquisition of the shock ability to use a more specific caspase inhibitor drug. Understanding the underlying molecular mechanism within NK cells that is responsible for secreting the LRA could perhaps be used to better exploit or boost the secretion of the LRA, thereby increasing the intensity of HIV-1 reactivation. Importantly, a recent study showed that human peripheral blood natural killer cells together with an LRA can efficiently delay HIV-1 rebound from latency following ART interruption and reduce the diversity of viral clones *in vivo* in a humanized mouse model, thus diminishing the HIV-1 latency reservoir (56). It will be interesting to implement our approach in such a humanized mouse model to assess the effects on the reduction of HIV-1 latency reservoirs. Altogether, our approach may be in combination with other shock-and-kill strategies or LRAs promising for reducing viral latency reservoirs and an important step forward towards the complete eradication of functionally active HIV-1 in people living with HIV-1.

## Data availability statement

The original contributions presented in the study are included in the article/**Supplementary Material**. Further inquiries can be directed to the corresponding author.

## Ethics statement

The studies involving human participants were reviewed and approved by the regional ethics committees of Stockholm (Sweden). The patients/participants provided their written informed consent to participate in this study.

## Author contributions

JM performed experiments, analyzed data, interpreted data and drafted the manuscript. LL, GG and LD performed experiments and analyzed data. LL and JPS contributed with their established 1C10 and PLA model. AS helped with scientific discussions, conceptualization of the project and funding acquisition. RD conceptualized the study, performed experiments, analyzed data, interpreted data, acquired funding, and drafted the manuscript. All authors critically revised, commented on, and approved the final manuscript.
