# Supplementary material for: Natural killer cells induce HIV-1 latency reversal after treatment with pan-caspase inhibitors
Source: Front Immunol. 2022 Dec 6;13:1067767. doi: 10.3389/fimmu.2022.1067767 (PMC9763267; doi:10.3389/fimmu.2022.1067767)
Supplement: Supplementary file 1 [file DataSheet_1.pdf]

Supplementary figures

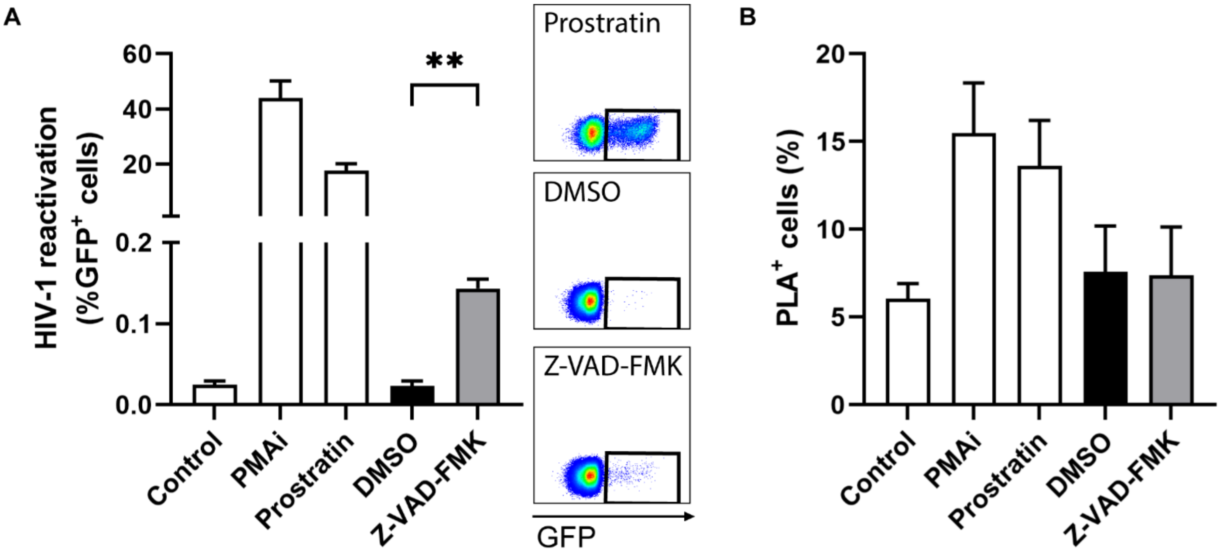

Supplementary figure 1.

1C10 cells were incubated with DMSO (control), PMA/ionomycin, prostratin, or supernatants of DMSO- or Z-VAD-FMK-treated KHYG-1 cells. After 16 h, cells were analysed by flow cytometry or PLA. **(A)** HIV-1 reactivations as measured by GFP-positivity was assessed by flow cytometry (n=3). Examples of gated GFP-positive cells are provided in pseudocolour plots. **(B)** LTR-bound Tat as measured by PLA-positivity was assessed by PLA (n=4). Data points are plotted as mean ± SD. (**$p < 0.01$; student T test)

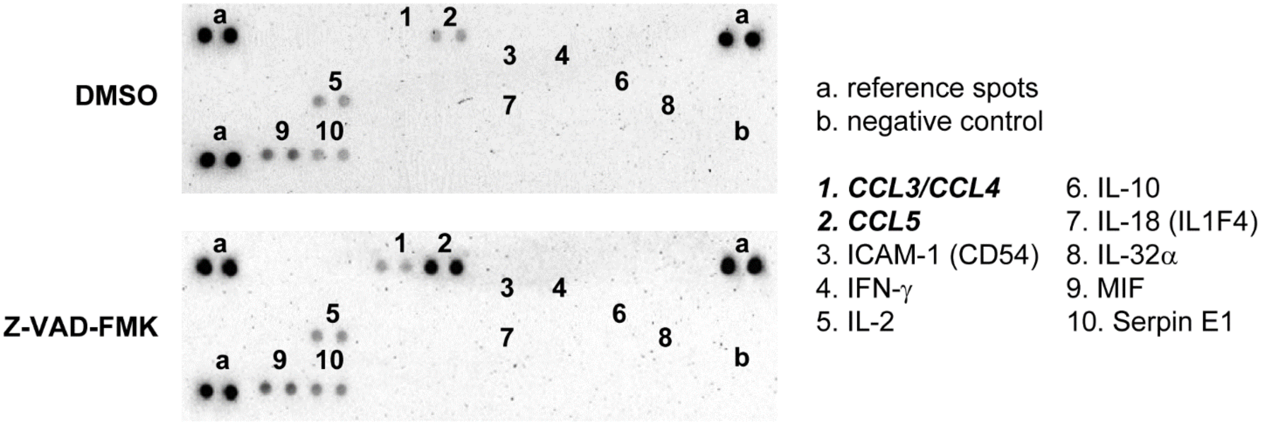

Supplementary figure 2.

Second cytokine array of supernatants from pan-caspase inhibitor-treated NK cells. KHYG-1 cells were incubated with Z-VAD-FMK or only DMSO. After 24 h, supernatants were collected and subjected to a cytokine array.

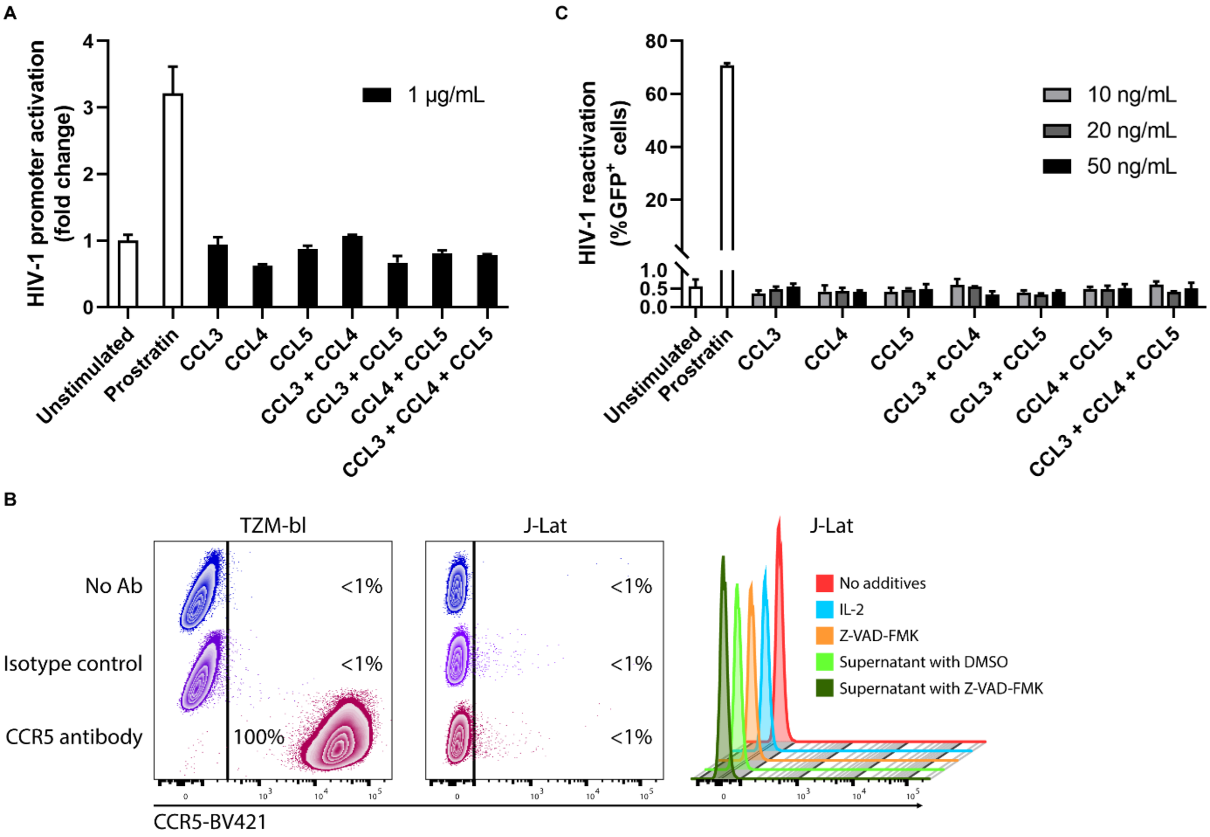

Supplementary figure 3.

CCL3, CCL4, and CCL5 do not induce an increase in HIV-1 promoter activity nor HIV-1 reactivation. **(A)** TZM-bl cells were incubated with high non-physiological concentrations (1 μg/mL) of CCL3, CCL4, CCL5, or combinations of these three chemokines (3 biological replicates). After 24 h, activation of the HIV-1 promoter was measured by luciferase assay. **(B)** J-Lat cells were cultured with various treatments for 24 h and cell surface expression of CCR5 was measured by flow cytometry. TZM-bl cells were used as positive control. Data points are plotted as mean ± SD. **(C)** J-Lat cells were incubated with various concentrations of CCL3, CCL4, CCL5, or combinations of these three chemokines (3 biological replicates). HIV-1 reactivations as measured by GFP-positivity was assessed by flow cytometry.

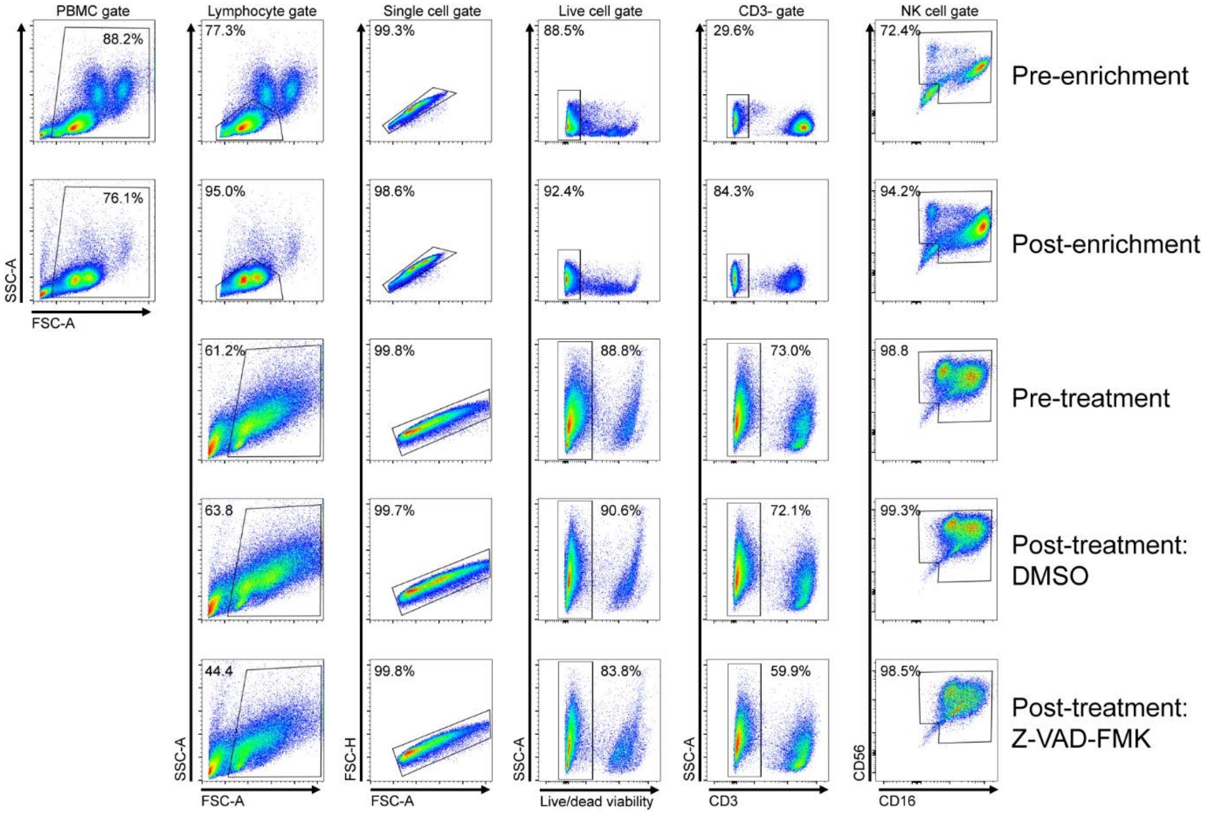

Supplementary figure 4.

NK cell distribution of the second donor. Flow cytometry analysis on semi-enriched primary NK cells from a second donor at different stages of the experiment; before semi-enrichment, after semi-enrichment, after 4 days of culture, and after 24 h treatment with DMSO or Z-VAD-FMK.

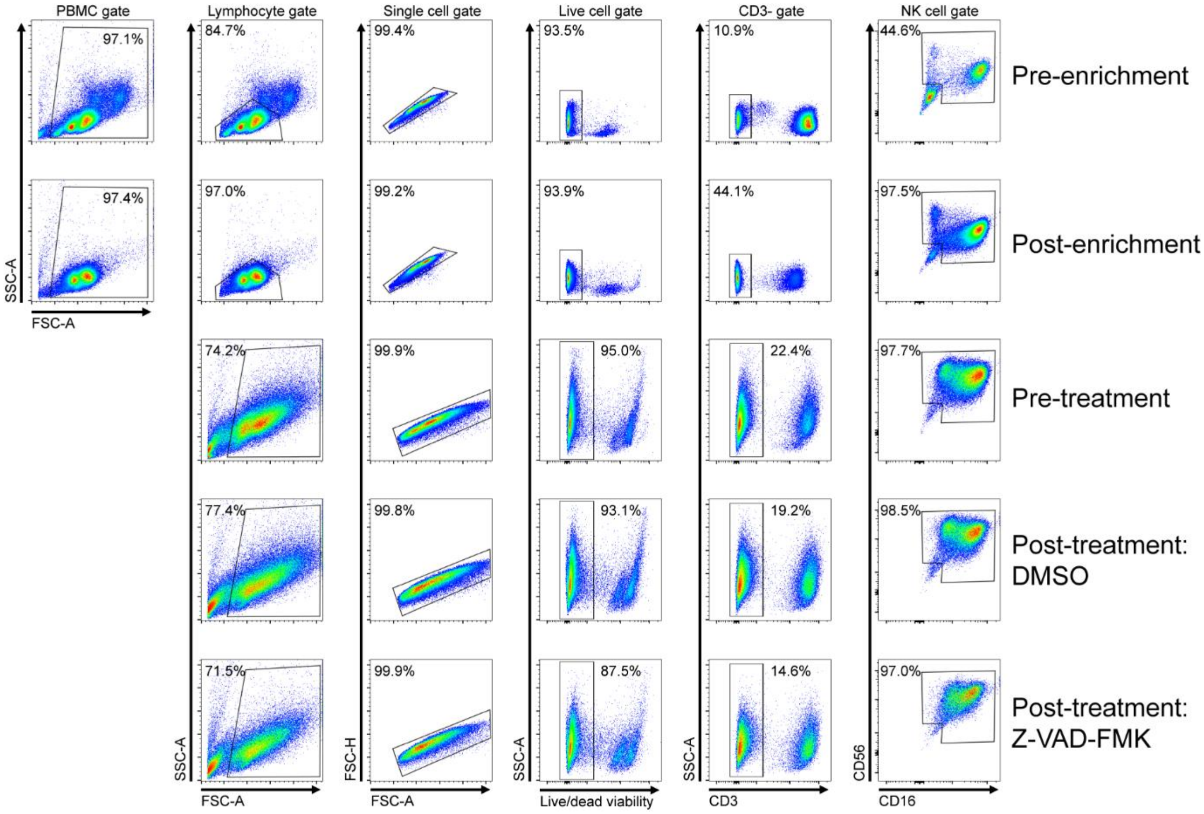

Supplementary figure 5.

NK cell distribution of the third donor. Flow cytometry analysis on semi-enriched primary NK cells from a third donor at different stages of the experiment; before semi-enrichment, after semi-enrichment, after 4 days of culture, and after 24 h treatment with DMSO or Z-VAD-FMK.

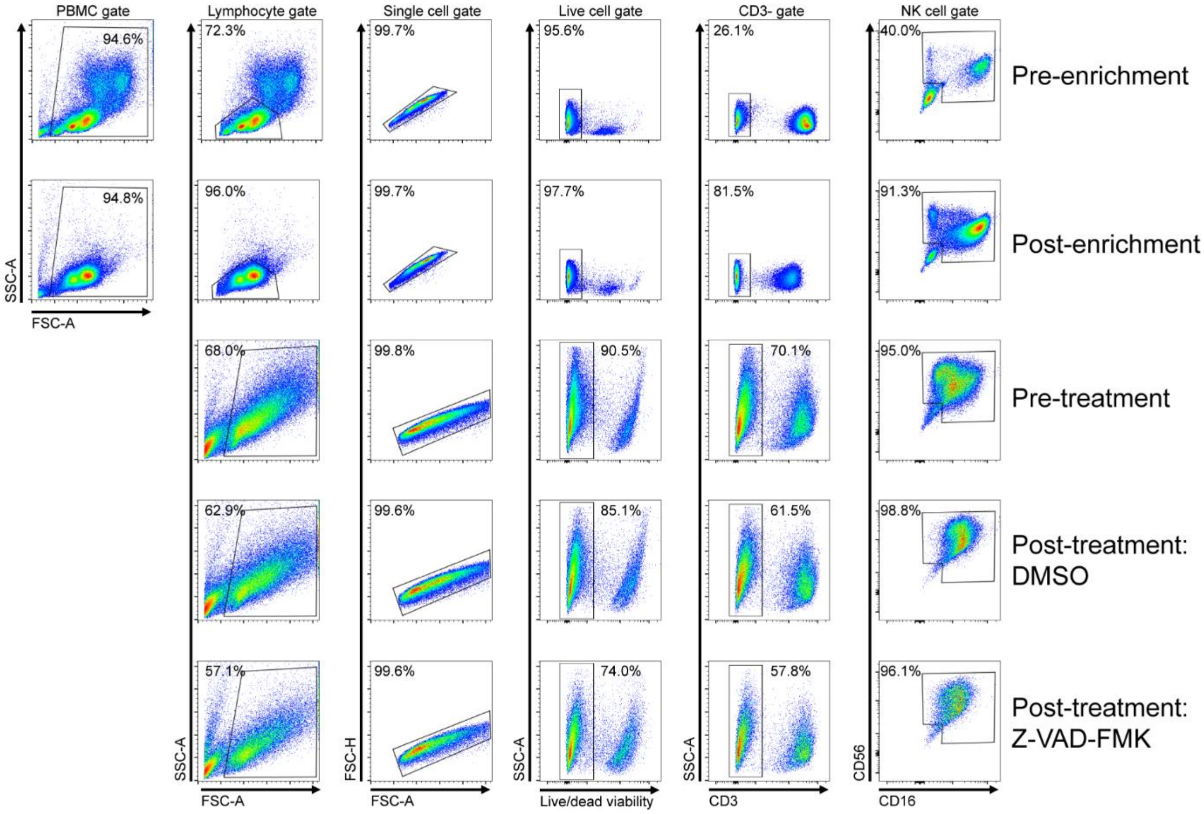

Supplementary figure 6.

NK cell distribution of the fourth donor. Flow cytometry analysis on semi-enriched primary NK cells from a fourth donor at different stages of the experiment; before semi-enrichment, after semi-enrichment, after 4 days of culture, and after 24 h treatment with DMSO or Z-VAD-FMK.
